# Velocity during Strength and Power Training of the Ankle Plantar and Dorsiflexor Muscles in Older Patients Attending Day Hospital Rehabilitation

**DOI:** 10.1155/2015/586843

**Published:** 2015-02-23

**Authors:** Pavithra Rajan, Michelle M. Porter

**Affiliations:** Faculty of Kinesiology and Recreation Management, University of Manitoba, Winnipeg, MB, Canada R3T 2N2

## Abstract

Power training has been proposed as a more effective type of resistance training for older adults for functional performance. It is not yet known whether older adults respond appropriately to instructions for power versus strength training. The purpose of this study was to determine the velocity during strength and power training, with elastic resistance bands, in older adults attending a geriatric rehabilitation day program. It was hypothesized that power training would be faster than strength training, but that there would be large interindividual differences. Nine older patients (70 to 86 years) performed power and strength training of the ankle dorsiflexor and plantar flexor muscles using elastic resistance bands. Training sessions were filmed to assess the velocity of training. Power training occurred at faster velocities as compared to strength training (*P* < 0.01) for both muscle groups. However, a wide variation was observed between participants in the training velocities. Older adults attending geriatric rehabilitation do have the potential to develop faster contractions during power training as compared to strength training. Nevertheless, the actual velocities achieved differed between individuals. This could explain some of the mixed findings of studies on power training. Hence, researchers should monitor velocity when comparing different types of resistance training.

## 1. Introduction

Several changes occur in the neuromuscular system with aging that result in weakness and loss of power [1]. Resistance training is advocated as a means of ameliorating the situation [1, 2]. Most of the early studies on resistance training for older adults have focussed on performing strength training, whereby high loads are moved at a relatively slow velocity [1]. These studies have demonstrated that older adults can experience muscle hypertrophy and also increase their strength.

However, it has been questioned as to whether strength improvements would translate into functional benefits, since most functional tasks require power more than strength [3]. Therefore, many investigators began exploring the benefits of power training as compared to strength training in improving strength, power, and function [4]. The results from many studies have been mixed, with only a slight overall benefit for power training over strength training in improving function, and the findings are not consistent [5–7]. One reason for the lack of difference between strength and power training could be that the velocity of training might not have been that different or different enough between groups. Only a few of the many studies of power versus strength training have actually measured or controlled the velocity or power output of training, and most often this has been done on highly sophisticated resistance training equipment such as Keiser pneumatic equipment [8, 9] or isokinetic dynamometers whereby velocity of movement was constrained [10]. With the equipment that has been more typically used for power training in older adults (e.g., weight machines, free weights, weighted vests, etc.), researchers, from the dozens of studies that have been done (as reviewed by [5, 6]), were reliant on the older adults to train at fast velocities, without knowing whether they were in fact doing so. Most researchers have used different verbal instructions for the concentric phase of movement (“as fast as possible” for power training and “slow and controlled” for strength training) (e.g., [11–13]). This has dominated the field so much that Tschopp et al. used “‘as fast as possible' movement speed” as a criteria for selecting power training studies from the literature in their meta-analysis comparing strength and power training [7].

During a previous power training study using resistance bands and weight machines in older mobility-impaired women [13], it was observed that even though individuals were given the same instructions for power training, they performed the contractions very differently (Webber and Porter, unpublished data). Hence, a pilot study was conducted with two participants from the published study who were performing power training exercises for the ankle using elastic resistance bands. They were videotaped and their average training velocities were analyzed using motion analysis software. While one participant trained at 20 and 37 degrees/second during dorsiflexion and plantarflexion training respectively, the other participant trained at 37 degrees/second and 80 degrees/second. So, despite giving similar instructions to the participants, they trained at different velocities. While the performance of the training movements were anecdotally observed to occur at a wide variety of velocities across all participants, that study, like many before it, did not include a measurement of the training velocity for all participants.

## 2. Purpose

The purpose of the current study was to examine whether older adults are able to train at different velocities when given instructions that are typical of strength and power training. It was hypothesized that power training contractions would be faster than strength training contractions, but that there would also be much interindividual variability within and between each type of training.

## 3. Materials and Methods

### 3.1. Study Design

In this study, all older participants enrolled performed both strength and power training of the ankle dorsiflexors (DF) and plantar flexors (PF). As the specific purpose was to examine the velocities during training, there were both within and between participant comparisons made. No pre- versus post-comparisons were done because the study was not designed to examine the outcomes of the training program, but rather* how* the participants trained.

### 3.2. Participants

Patients 65 years and older attending a day hospital geriatric rehabilitation program were eligible to participate in the study if they were: cognitively intact (e.g., could follow instructions for strength and power training as assessed by program staff based on their clinical judgment and patient files where cognition was recorded if there were concerns and it was measured), were able to participate in exercise (e.g., did not have any unstable heart conditions), and particularly were able to do ankle exercises, had not participated in any strength or power training of the PF and DF muscles in the past 6 months, did not have a systemic neural condition that majorly affected movement control (e.g., Parkinson's disease or multiple sclerosis), and would be attending the day hospital program long enough for them to complete the training sessions. All patients were first approached by staff at the geriatric rehabilitation program based on them generally meeting the eligibility criteria. Next, the authors met with those who were interested in hearing more about the study to (1) explain the study in a detailed way; (2) determine whether they were still interested in participating; and (3) determine whether they met all the inclusion/exclusion criteria. Thirteen participants were informed about the research study by program staff and met with the researchers. One potential participant was excluded from the sample due to possible cognitive issues that did not allow her to provide informed consent. Out of the remaining 12 potential participants, one developed acute swelling of a leg and so could not participate, another was not able to make transportation arrangements to attend the program, and the final person who was not able to participate fully was hospitalized after his first session. This left nine participants who completed the study.

Given that this was done with patients attending a day hospital, the participants did have many medical conditions and were typically recovering from an illness (pneumonia), stroke, joint replacement, and so forth, although after initial treatment or rehabilitation for their very specific medical event. All participants provided written informed consent prior to the study. Ethical approval was obtained from the Education/Nursing Research Ethics Board at the University of Manitoba, and Riverview Health Centre, Winnipeg, Canada.

### 3.3. Training Protocol

All training was done with elastic resistance bands (Thera-Band, The Hygenic Corporation, Akron, Ohio), because they are portable and inexpensive pieces of equipment that were already in use in this rehabilitation setting. During the first session, ankle DF isometric strength was measured using a hand held dynamometer with the participant comfortably seated on a standard chair with 90 degrees of flexion at hip and knee (MicroFet2 MT, Hoggan Health Industries, Utah). This type of test has been found to be reliable in older adults [14]. Given the rehabilitation setting in the day hospital and the equipment available, it was not possible to test the strength of dynamic contractions as well as the strength of PF.

Based on DF strength, bands were chosen for the DF and PF for each individual, with the bands for PF training having a higher resistance level than the band chosen for DF training. This was followed by several familiarization trials of strength and power training for both the DF and PF. During the second session, the order of exercises was randomized for each participant. The same order of exercises was followed for subsequent sessions, for the particular participant. From the second session onwards, each participant performed 8 repetitions each of DF and PF strength and power training (4 sets in total). Each of the 32 movements at each training session was video recorded for later analyses. For power training, the participants were instructed to perform the ankle movement “as fast as possible” during the concentric phase of the exercise, whereas for strength training, the participants were instructed to perform the ankle movement in a “controlled” way throughout the available range of motion as is typically done for strength training. For both power and strength training, the participants were instructed to perform the eccentric phase in a controlled fashion. Both legs were individually trained in all participants alternately so that while one leg trained, the other leg was resting. One Certified Exercise Professional individually supervised all training sessions and gave specific instructions for each contraction (e.g., for power training: “fast up, controlled down”).

### 3.4. Velocity Assessment

**Figure 1:**
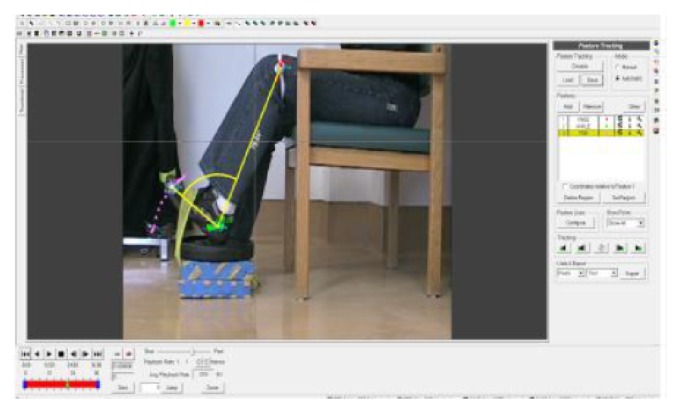
A sample is shown of one of the investigators performing a dorsiflexion contraction, within the software used to analyze the repetitions.

Only one side was filmed for each participant. The participants were filmed using a digital camcorder (Canon 200MC Optura mini DV camcorder, Tokyo, Japan) which had a sampling rate of 30 frames per second. It was placed approximately 2 metres lateral to the filming leg, in order to capture a sagittal view of the ankle movement. The camera was affixed to a tripod (T120 Minipro Tripod, Optex, New Jersey, USA). In order to identify the hip, the knee, and the lateral border of the foot, markers were used. Markers were stuck onto the pants and the shoes of the participants at the hip, the ankle, and the toe. An elasticized band was used for marking the knee. The position of the markers was measured with respect to bony landmarks and the markers were placed at the same place every time the participant came to exercise. See Figure 1 for an example of the set up.

Video files were analyzed using Proanalyst Professional Edition—Version 1.5.4.0 (Xcitex, Cambridge, USA)—to determine the angular velocity for each repetition. The main outcome variables were peak angular velocity and median angular velocity. Since angular velocity (only velocity will be used in future for brevity sake) was not normally distributed in every repetition, the median velocity was used to provide a representative velocity for each repetition instead of average velocity.

The specific reliability of making the measurements of velocity (median and peak) in this study was assessed in a subsample of data. Measurements of interest were made using several repetitions of four participants, and then these measurements were made again after a 1-week interval. It is important to note that the process for making the measurements is semi-automated such that the markers are identified by the operator, but the actual angular velocity is determined by the software as it “follows” the markers through their trajectories (PF and DF). It was found that the measurements for median and peak velocity were reliable (coefficients of variability ranged from 3.6% to 8.9% for DF variables and 3.9% to 11.5% for PF for the variables of interest in this study—median and peak velocity, resp.).

### 3.5. Statistical Analyses

Statistical analyses were performed using SigmaPlot (version 11, Systat Software, Inc, San Jose, CA). In order to examine changes in peak and median velocity between repetitions within a set, one way Repeated Measures ANOVA were used. Since there was no consistent pattern within a set (i.e., the repetitions did not get slower as more repetitions were done), all repetitions from all training sessions were used in calculating the mean values for median and peak velocities for further analyses. To determine if there were differences between strength and power training in terms of velocity (peak and median), separate paired *t*-tests were conducted.

## 4. Results

### 4.1. Participants

See Table 1 for the participant characteristics. The nine participants of this study ranged in age from 70 to 89 years. Seven out of 9 participants were women. All participants except one used walking aids. All except three participants had a fall in the past year. There was only one participant who did not suffer from any long term conditions. The long term conditions that the participants suffered from included high blood pressure, arthritis, heart disease, neck and back problems, cancer, osteoporosis, fibromyalgia rheumatic, stroke, and pernicious anemia.

### 4.2. Training Velocities

For the group, the paired *t*-test analyses indicated that for both DF and PF there were significant differences (*P* < 0.01; see Table 2) between strength and power training, regardless of whether median or peak velocities were used. In all cases, power training was faster than strength training.

**Figure 2:**
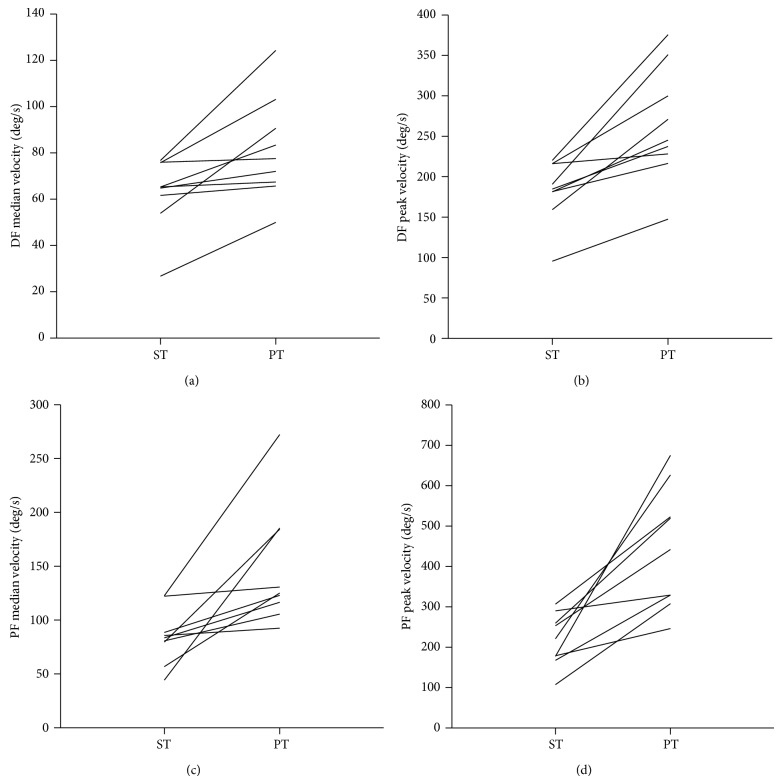
Individual data (i.e., each line is an individual) are shown for strength training (ST) and power training (PT) for (a) dorsiflexion (DF) median velocity; (b) DF peak velocity; (c) plantar flexion (PF) median velocity; and (d) PF peak velocity.

When examining data for the individual participants, a wide range of training velocities was observed (see Figure 2). For DF, median velocities ranged from 26.7 to 76.7 degrees/second for strength training, and from 50.0 to 124.2 degrees/second for power training. For PF, median velocities ranged from 44.1 to 122.6 degrees/second for strength training, and for power training from 92.4 to 272.3 degrees/second. A similar level of variability was seen for peak velocity values. Also as demonstrated in Figure 2, there was overlap between the velocities during strength and power training of both the DF and PF between participants. This meant that some participants trained at velocities in strength training that were faster than the power training velocities of other participants. In terms of differences between strength and power training velocities for individuals, there were small (2.1%) to large differences (319.8%). For some individuals, this meant that power training was just barely faster, and for others it could be up to 3 times faster than strength training.

## 5. Discussion

The purpose of this study was to determine if there were any differences in the training velocities when using elastic resistance bands for strength and power training during dorsiflexion and plantar flexion in older patients attending a day hospital rehabilitation program. In this study, power training occurred at higher velocities as compared to strength training for both DF as well as PF. Studies to date have not typically measured the velocity during strength and power training. Apparently, it has been assumed that when older adults are instructed to perform movements “as fast as possible”; they would perform the movements at higher velocities.

In a previous study of power training [13], it was observed that the older mobility-impaired women participating were quite variable in their velocity of training (unpublished data), which precipitated this project. In the current study, objectively measured velocities between individuals had a large range even though they were highly supervised and given very specific instructions for each contraction performed. This resulted in some individuals having overlapping velocities between strength and power training (i.e., some individuals trained at faster velocities during strength training than others used for power training). In addition, some individuals had only having minor differences between strength and power training velocities, whereas one individual had velocities that were 3 times higher during power training than strength training. It is unknown what this would mean for the efficacy of a power training program. For example, would the individuals who train at lower velocities have lower responses to the training than those who train at high velocities? Further research is needed with well-designed randomized trials in determining how training velocities affect outcomes of training. Also, research should be done exploring what factors, including age-related neuromuscular factors and health conditions, might be contributing to the velocities that older adults choose to use when performing strength and power training.

Intervention studies have used high speed training in older adults with promising results [4–7]. These power training studies have used a variety of different types of equipment including: weighted vests, free weights, and weight machines [5, 6]. Benefits of power training have ranged from increases in strength and power to physical performance (e.g., chair rise, stair climbing) [6]. Additional benefits that have been reported are increased muscle volume and muscle mass [7] and improved balance [5–7]. However, not all findings are consistent, as Steib et al. conclude in their meta-analysis of dose response in resistance training, “further studies are necessary to clearly point out the benefits of PT (power training)” in terms of improving physical performance because there were inconsistent findings on whether power training was more effective than strength training. We suggest that researchers specifically monitor the velocity of training because it could be a factor in explaining the inconsistent results.

One study that did measure velocity during power versus strength training was Reid et al. [9]. They found that those who power trained attained velocities that were over twice the velocities attained during strength training for knee extension and leg press, as measured by the pneumatic resistance training equipment that was used. Fielding et al. [8] examined power output during training and found that it was two to almost four times higher in high velocity training as compared to low velocity training. Only average data was provided for the two studies mentioned [8, 9]. Fielding et al. measured power output rather than velocity [8], and different muscle groups were exercised, so it is difficult to compare their study with the current study. Like Reid et al. though, PF power training peak velocity was over twice as fast as strength training peak velocity for our group of participants. Surprisingly and contrary to their hypothesis, Reid et al. did not find greater training-induced improvements in power generation for the power training group than the strength training group [9]. They suggested that further studies are needed to determine the optimal resistance training parameters for aiding older adults to improve strength, power and functional performance. This sentiment was echoed by recent meta-analyses [6, 7], which have found inconsistent findings in whether strength training or power training improved functional performance more.

## 6. Limitations

One limitation of the current study is that the participants were novice trainers. A possible reason for the inability of performing the requested movements at different speeds might be attributed to the relative inexperience of the individuals. Trainers with more experience might have developed better force control and be able to perform the movements without large variability because they were allowed to learn more. However, we did not find that velocity of training changed over the sessions attended in this study. In our previous study, we anecdotally observed that the difference in training velocity persisted across the whole 12 weeks of the study [13]. In addition, most resistance training studies reported in the literature involve older adults who are inexperienced. Future studies could examine inexperienced and experienced trainers to see what velocities are used in power training.

Another possible limitation of the current study involves the loads used for training. Although different loads have been used in the reported studies [6], the American College of Sports Medicine recommends that power training should be performed with lower loads and higher movement velocities in comparison to regular strength training where loads are high and movement velocity is “controlled” [1]. In this study, older adults did not use different loads for strength training versus power training, and because bands were used it was not possible to prescribe specific loads relative to their maximum strength or power. Even though the loads were the same, we did see significant differences between strength and power training at the group level. At the individual level some of the variability might have been caused by the inability to judge the exact load to use, and the fact that with resistance bands, there is a change in the loading pattern due to the lengthening of the bands. This makes it hard to compare the results of this study with conventional resistance training machines that control for these load variations. In our previous study [13] we did observe that training velocities did vary even when participants were using weight machines and repetition maximum testing was done to prescribe the load more precisely (unpublished data). Future studies examining velocities during training should use loads that can be more accurately quantified and prescribed.

## 7. Conclusions

While both strength and power training are forms of resistance training that have many benefits, it is not clear whether strength training or power training can deliver different outcomes for older adults. One complication to this type of research is how the training is done. With the common types of equipment that are used, there is no way to control the movement velocity, which is the essence of power training. In this study, we examined whether older adults attending a day hospital rehabilitation program would be able to train at faster velocities during power versus strength training. They did perform contractions of both the DF and PF at faster velocities during power training than strength training. However, there was a large range of velocities used across the participants. Future research should examine the individual responses to power and strength training while examining how the training was actually performed. This might provide insights for the types of benefits that can be expected with these forms of resistance training.

## Figures and Tables

**Table 1:** Participant characteristics.

Characteristic	Descriptive statistic
Age (years)	82 ± 6 (Mean ± SD)
Gender (% male)	22.2
Use of walking aids (%)	88.9
Had a fall in the past year (%)	66.7
General health for age	Good (median)
Medications taken in the past month (#)	Two (median)
Chronic conditions per person (#)	3
Cardiovascular disease and/or high BP (%)	55.5
Arthritis or bone or other joint problems (%)	77.8

**Table 2:** Velocities (degrees/second) during dorsiflexor (DF) and plantar flexor (PF) strength and power training.

	Strength training	Power training
DF median	62.9 ± 15.5	81.5 ± 22.2^*^
DF peak	182.8 ± 38.5	263.5 ± 70.3^*^
PF median	84.8 ± 25.8	148.3 ± 56.5^*^
PF peak	217.9 ± 65.2	444.3 ± 151.2^*^

Note: ^*^power training significantly faster than strength training (*P* < 0.01).
